# The Relationship Between Loneliness and Academic Stress: The Mediating Role of Lack of Academic Organization Among Social Work Students in Chile

**DOI:** 10.3390/bs16081439

**Published:** 2026-08-20

**Authors:** Guillermo Davinson-Pacheco, Julio Tereucan-Angulo, José Luis Gálvez-Nieto, Ignacio Norambuena-Paredes, Vicenta Rodríguez-Martín, Marisol Lemunao-Díaz

**Affiliations:** 1 Departamento de Trabajo Social, Universidad de La Frontera, Temuco 4780000, Chile; guillermo.davinson@ufrontera.cl (G.D.-P.); jose.galvez@ufrontera.cl (J.L.G.-N.); ignacio.norambuena@ufrontera.cl (I.N.-P.); 2 Facultad de Ciencias Sociales y Tecnologías de la Información, Universidad Castilla la Mancha, 28001 Talavera de la Reina, Spain; vicenta.rodriguez@uclm.es; 3 Programa de Doctorado en Intervención Psicosocial, Universidad de La Frontera, Temuco 4780000, Chile; m.lemunao01@ufromail.cl

**Keywords:** academic stress, lack of academic organization, loneliness, social work education, social work training

## Abstract

The study of loneliness and academic stress is a priority area in university research, given their impact on student well-being, retention in higher education, and academic performance. In Chile, with respect to Social Work education, these phenomena acquire relevance, given that students simultaneously face high curricular demands and professional training practices in contexts of social vulnerability. In this context, a lack of academic organization may contribute to the perception of overload and limit students’ academic coping strategies. The objective of this study was to examine the relationships between loneliness, lack of academic organization, and academic stress among Social Work students in Chile. A total of 298 students from nine public and private universities participated, with an average age of 21.74 years (SD = 3.47). The results, obtained through a structural equation model, confirmed that the relationship between loneliness and academic stress was partially mediated by a lack of academic organization; however, the direct association between loneliness and academic stress represented the strongest effect within the model. Likewise, positive direct effects were observed among the three variables. Taken together, these findings reinforce the need to design educational and institutional strategies oriented at promoting student well-being, thereby strengthening the perception of professional preparedness among future social workers.

## 1. Introduction

Academic stress has emerged as a central construct in educational and psychosocial research, given its direct impact on academic performance, mental health, and retention in higher education (Chust-Hernández et al., 2023; Peris-Ramos et al., 2025; Ueno et al., 2025). In the field of Social Work education, its analysis takes on relevance, as students simultaneously face high curricular demands, professional training practices in contexts of social vulnerability, and institutional demands that intensify their educational experience (Afrouz & Crisp, 2021; Baird, 2016; Motloung & Mzinyane, 2023; Pham et al., 2023).

In this context, within postmodern society characterized by the fragmentation of social ties, the acceleration of cultural changes, and the centrality of individual autonomy, new forms of vulnerability are emerging that directly impact the student experience (Han, 2014; Norambuena-Paredes, 2025). Self-exploitation, constant competition, and the individualized management of academic success place young people in a scenario of increasing demands that weaken community bonds (Ball et al., 2024; Sing-Yun, 2023). These processes of individualization, described by Beck and Beck-Gernsheim (2002), force students to face their university journey under a heavy personal burden, in a context marked by both curricular demands and sociocultural tensions that affect their well-being and life satisfaction (Gálvez-Nieto et al., 2025; Norambuena-Paredes et al., 2025).

Within the field of Social Work, international organizations such as the International Federation of Social Workers (IFSW) and the International Association of Schools of Social Work (IASSW) have emphasized the importance of strengthening wellbeing and self-care during professional training. Nevertheless, in many university contexts, these dimensions are not always explicitly integrated into curricula, despite the considerable academic, emotional, and practical demands that characterize Social Work education (Afrouz & Crisp, 2021; Baird, 2016; Kangasniemi et al., 2022). In this context, phenomena such as loneliness and academic stress become particularly relevant, as they may negatively affect students’ mental health, academic performance, and educational trajectories (Brown et al., 2018; Campagne, 2019; Real-Delor et al., 2024; Ueno et al., 2025).

In this regard, loneliness in the university setting can be understood as an unpleasant experience stemming from dissatisfaction with the available system of personal and social relationships (González-Casas et al., 2022). This approach highlights its subjective nature by focusing on the perception of insufficient or fragile support networks (Sedláková & Křeménková, 2019; Vicary et al., 2025). However, its scope transcends the individual sphere and is rooted in transformations characteristic of late modernity. Authors such as Bauman (2003) warn that processes of individualization, the fragmentation of community ties, and the centrality of personal autonomy have eroded traditional structures of social support, generating new forms of vulnerability. Thus, university loneliness should not be conceived solely as a relational deficit, but as a complex phenomenon that articulates psychosocial and structural dimensions, significantly impacting students’ academic well-being (Södel et al., 2025).

From a psychosocial and critical perspective, this study is grounded in the concepts of liquid modernity and contemporary processes of individualization (Bauman, 2003; Beck & Beck-Gernsheim, 2002), which provide a framework for understanding how the fragility of social bonds, increasing demands for autonomy, and pressures associated with performance shape new forms of vulnerability among university students. Within this framework, loneliness, academic disorganization, and academic stress are understood not merely as individual phenomena, but also as expressions of structural tensions embedded in the contemporary university experience.

In parallel, the lack of academic organization constitutes a structural factor that complicates the university experience (Vasquez et al., 2023; Vieyra et al., 2024). This dimension is related to behaviors such as a lack of study materials, forgetting previously covered content, unexcused absences from classes, late submission of assignments, and reduced rest hours due to last-minute studying (Preciado-Serrano et al., 2021). Such manifestations reflect not only individual deficits in learning self-regulation, but also institutional limitations in the provision and organization of pedagogical support (Romero-Pérez & Sánchez-Lissen, 2022; Wolters & Brady, 2021). In this sense, academic disorganization should not be understood exclusively as a purely personal variable, as it is intertwined with the structural conditions of the university environment (Y. Wang, 2015).

Empirical evidence suggests that this dimension has a significant effect on academic stress, as it increases the perception of overload and reduces the ability to cope with educational demands (Gao, 2023; Qian & Fuqiang, 2018). The absence of adequate planning and sufficient pedagogical resources creates a constant sense of uncertainty among students, who find their range of action limited when it comes to effectively meeting curricular demands (Peker, 2024; Wolters & Brady, 2021). This scenario is intensified in fields such as Social Work, where the combination of theoretical and practical activities requires solid organization of educational processes to ensure the integration of knowledge and the development of professional competencies (Boladeres et al., 2024; Boynton, 2025; Kangasniemi et al., 2022).

Likewise, the interaction between loneliness and a lack of academic organization provides a broader understanding of the predictors of academic stress (Jefferson et al., 2023; Liu & Li, 2023). Although distinct in nature, both dimensions converge on the same outcome: the weakening of the psychosocial and pedagogical support mechanisms that allow students to sustain a successful university experience (Ball et al., 2025; Moriña & Biagiotti, 2022). While loneliness weakens the relational fabric that provides emotional support and a sense of belonging, academic disorganization limits access to basic conditions for structured learning (Ellard et al., 2023). Taken together, these factors may contribute to a dynamic in which demotivation, overload, and isolation tend to reinforce one another, increasing student vulnerability and academic stress among college students (Aslan et al., 2025; Norambuena-Paredes et al., 2025; Troncone et al., 2025).

In this line, recent research has shown that academic stress should be understood as a multidimensional phenomenon in which relational, pedagogical, and contextual factors converge. The American Psychiatric Association (2023) warns that the combination of social isolation and institutional demands acts as a powerful trigger for mental health problems among the university population. Complementarily, the World Health Organization (WHO, 2022) has indicated that the absence of support networks and structured educational environments weakens retention and equity in higher education (Harris et al., 2022). These perspectives reinforce the need to examine how loneliness and lack of academic organization may jointly contribute to conditions of vulnerability, the understanding of which is essential for designing evidence-based prevention and intervention strategies.

### 1.1. The Relationship Between Loneliness and Academic Stress

Loneliness, understood as the perception of emotional or social disconnection, has been identified as a significant contributing factor to academic stress among college students (Korzhina et al., 2022; Niazov et al., 2022; Walsh et al., 2025). In educational contexts with high academic demands, a lack of social support and perceived exclusion can generate a sense of isolation that exacerbates pressures related to academic performance (Dominguez-Lara et al., 2024; Lei et al., 2022; San & Guo, 2023). Research suggests that loneliness not only increases vulnerability to stress but also interferes with the coping strategies students use to manage their academic responsibilities (Hamdan-Mansour et al., 2025; Quan et al., 2014; Zhang et al., 2025). The lack of social support networks can limit opportunities for emotional validation, creating a negative cycle that reinforces both the perception of loneliness and the stress associated with academic workload (Melaku et al., 2025; Stasolla et al., 2025).

The scientific literature has shown that perceived loneliness influences students’ emotional well-being, which translates into a higher risk of experiencing symptoms of academic stress, anxiety, and depression (Brunsting et al., 2021; Hadad, 2025; Özdoğan, 2021; Yani et al., 2025). This phenomenon is particularly evident in situations where students, feeling disconnected from their classmates or professors, develop a greater sense of maladjustment in the face of academic demands (Benítez-Agudelo et al., 2025; Ueno et al., 2025). The inability to share experiences and concerns with others generates a direct impact on students’ ability to manage academic pressure, leading to a decline in performance and an increase in emotional exhaustion (Jiang et al., 2025; Klinkenberg et al., 2024).

On the other hand, the interaction between loneliness and academic stress is influenced by individual factors such as self-esteem and resilience. Students with low levels of self-efficacy and coping skills are more likely to experience the negative effects of loneliness, which reinforces the relationship between the two factors (Malakcioglu, 2024; Y. W. Wang et al., 2024). Likewise, the perception of control over the academic situation is diminished when students feel lonely, which increases the sense of stress (Benítez-Agudelo et al., 2025). This context highlights the importance of interventions focused on strengthening social support networks and on developing social–emotional competencies as effective strategies for mitigating both loneliness and academic stress in university settings (Walsh et al., 2025).

In summary, the relationship between loneliness and academic stress is bidirectional, where loneliness increases stress and, in turn, stress intensifies the feeling of isolation (Brown et al., 2018; Campagne, 2019). Understanding this dynamic is crucial for designing social and emotional support strategies that not only address the immediate causes of stress but also foster a greater social integration within the academic setting.

### 1.2. The Relationship Between Loneliness and Lack of Academic Organization

Loneliness in the university setting is a subjective experience that goes beyond the mere absence of social relationships, manifesting itself as an experience of emotional and symbolic disconnection from others and from the educational institution (Ellard et al., 2023; Norazizi et al., 2024). Various studies have indicated that this condition negatively impacts the processes of self-regulation of learning by weakening the interpersonal support that facilitates the planning, monitoring, and evaluation of academic tasks (Iwasaki et al., 2025; Tadesse et al., 2024). In this sense, loneliness can erode the psychosocial resources necessary to sustain stable study routines, favoring disorganization dynamics, which manifest themselves in difficulties prioritizing tasks, managing time, and maintaining continuity in academic work (Alinejad et al., 2022; Potter et al., 2025).

From a psychosocial perspective, the relationship between loneliness and poor academic organization can be understood as a bidirectional and cumulative process (Hamdan-Mansour et al., 2024; Leich et al., 2025). On one hand, the lack of meaningful connections with peers and teachers limits the access to key informal information such as reminders, practical orientation, and shared study strategies, which typically serves as an organizational framework in university life (Li et al., 2024; Lin & Fan, 2023). On the other hand, a lack of academic organization reinforces the feeling of isolation, as missing deadlines, skipping classes, or low participation in educational activities tend to further weaken the student’s social and academic integration (López-García et al., 2023; Umerenkova & Flores, 2022).

Likewise, loneliness can intensify the perception of cognitive and emotional overload, affecting the ability to plan academic tasks in advance (Ostrow et al., 2025; Uyaroğlu et al., 2022). Recent research suggests that students who report higher levels of loneliness experience greater difficulties with executive functions related to organization, such as planning and attention control, which results in improvised and inefficient study strategies (Hussin et al., 2021; Troncone et al., 2025). In this scenario, the situation is particularly critical in educational contexts characterized by high academic demands and limited personalized pedagogical guidance, where the required autonomy is not always accompanied by sufficient institutional support (Stead et al., 2025).

Lastly, understanding the relationship between loneliness and a lack of academic organization requires recognizing that both factors are rooted in structural conditions within the university environment and are not exclusively individual in nature. The fragmentation of educational trajectories, the massification of higher education, and the limited availability of academic and psychosocial support spaces can simultaneously deepen student disorganization and isolation (Kulari et al., 2026; Wenig et al., 2025). Consequently, addressing this issue requires comprehensive strategies that articulate pedagogical interventions, social–emotional support mechanisms, and institutional policies to strengthen students’ academic and social integration, thereby contributing to a more organized, inclusive, and sustainable university experience.

### 1.3. Relationship Between Lack of Academic Organization and Academic Stress

A lack of academic organization has been identified as a significant factor in the intensification of academic stress, particularly in university settings characterized by high demands and overlapping deadlines (Akyürek, 2021; Brambila-Tapia et al., 2025). Difficulty in planning study, managing time, and anticipating assessments increases the perception of a lack of control over academic demands and is one of the main triggers of stress among higher education students (Brambila-Tapia et al., 2025; Salguero-Pazos & Reyes-de-Cózar, 2023). This experience of cognitive overload tends to generate a constant sense of urgency and pressure, affecting psychological well-being and academic performance (Stasolla et al., 2025).

From a cognitive perspective, academic disorganization interferes with the processes of self-regulation in learning, undermining the ability to set clear goals, monitor the learning process, and adjust study strategies (Inquilla-Mamani et al., 2025; Salari et al., 2025). When these skills are compromised, academic tasks are often approached in a reactive and improvised manner, which increases the mental and emotional burden associated with studying (Calle-Müller & ElZomor, 2025; Wijbenga et al., 2024). In this context, academic stress arises not only from the sheer volume of academic demands but also from the perception of an inability to respond to them adequately (Estrada-Araoz et al., 2024; Gutierrez et al., 2025).

Likewise, a lack of academic organization is associated with behavioral patterns that deepen stress, such as procrastination of tasks, study cramming for brief periods, and the reduction in rest and sleep hours (Behera et al., 2025; Mojibpour et al., 2025). The accumulation of pending tasks and missed deadlines reinforces feelings of anxiety, guilt, and exhaustion, creating a vicious cycle that is difficult to break without social support (Patzak et al., 2025).

Despite advances in the understanding of academic stress, important gaps remain regarding the identification of specific variables that contribute to this phenomenon in highly demanding educational contexts, such as Social Work training. Much of the existing literature has focused on individual or emotional factors, while giving less attention to the interaction between relational dimensions, such as loneliness, and structural dimensions, such as a lack of academic organization. Exploring this relationship is particularly relevant, as it allows us to move beyond reductionist perspectives and towards a more comprehensive understanding of student distress.

In this regard, the relationship between a lack of academic organization and academic stress should also be understood as a phenomenon associated with institutional conditions (Chust-Hernández et al., 2024). Limited clarity in course structures, excessive assessment demands, and insufficient coordination between modules may intensify students’ academic disorganization and, consequently, academic stress (Islam & Rabbi, 2024; Qian & Fuqiang, 2018). Considering this background, the present study seeks to address existing gaps in university research on the factors associated with academic stress, particularly within the context of Social Work education, by examining whether a lack of academic organization partially mediates the relationship between loneliness and academic stress among Social Work students in Chile.

Accordingly, the present study seeks to answer the following research question: How are loneliness, lack of academic organization, and academic stress related among Social Work students in Chile? In this context, the general objective of the study is to examine the relationships between loneliness, lack of academic organization, and academic stress in a sample of Social Work students in Chile. In doing so, the study aims to provide empirical evidence that contributes both to scientific debate and to the development of educational policies and strategies aimed at strengthening student well-being and retention in higher education.

**Figure 1:**
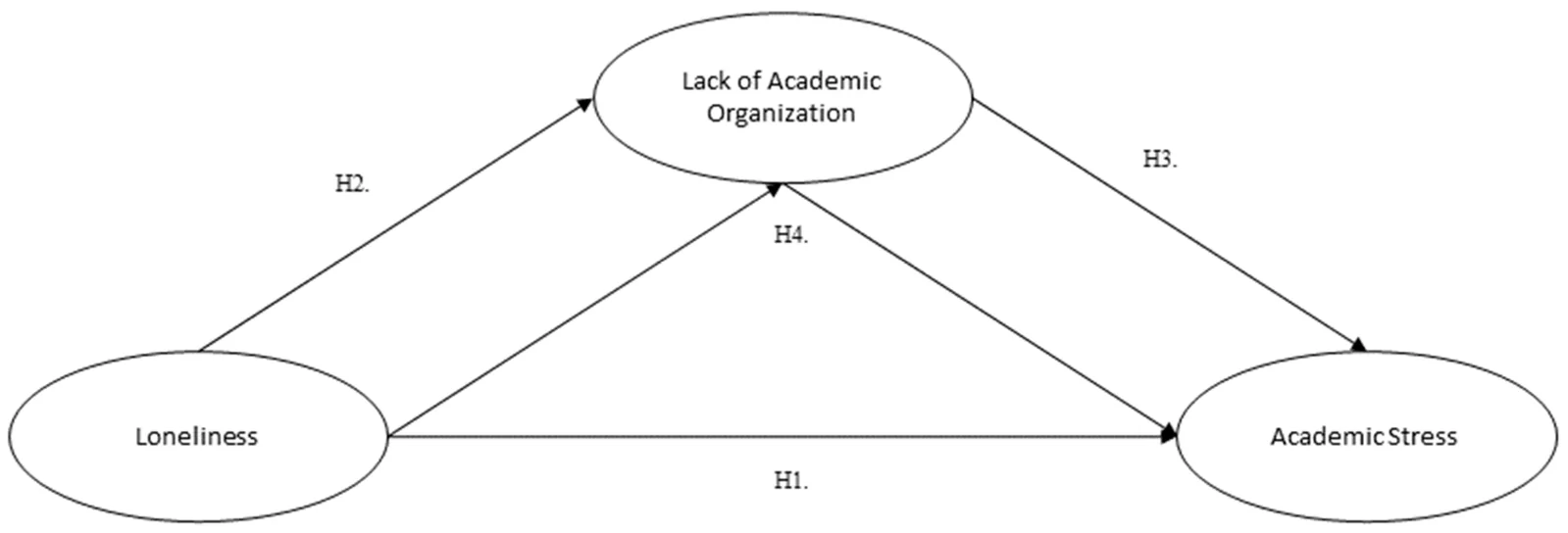
Theoretical diagram of the relationships between variables. Note. Single-headed arrows indicate the direction of the hypothesized effects between variables. H1–H4 correspond to the proposed hypotheses of the mediation model.

Although previous research has demonstrated associations between these constructs, empirical evidence simultaneously examining their direct and indirect relationships remains limited. Based on this objective, four hypotheses are proposed and presented in Figure 1: (H1) Loneliness is positively associated with academic stress. (H2) Loneliness is positively associated with a lack of academic organization. (H3) A lack of academic organization is positively associated with academic stress. (H4) The relationship between loneliness and academic stress is mediated by a lack of academic organization.

## 2. Materials and Methods

The present study adopted a quantitative, correlational approach with a cross-sectional design to examine the relationships between loneliness, lack of academic organization, and academic stress among Social Work students in Chile. This methodological approach enabled the simultaneous analysis of both direct and indirect associations between the study variables within a specific temporal context.

### 2.1. Participants

The study population consisted of undergraduate students enrolled in Social Work programs from nine public and private universities in Chile. A stratified sampling strategy was implemented, considering each university as a stratum. Sample allocation was proportional to the number of enrolled students at each institution, following the parameters proposed by Scheaffer et al. (2000) for finite populations (95% confidence level, 5% margin of error, and *p* = q = 0.5).

Participation was voluntary and anonymous. Data collection was coordinated with program directors and academic staff at each institution. Trained Social Work students administered the questionnaire during regular class sessions using the QuestionPro platform. Therefore, although the study was designed under a stratified probabilistic framework, participation ultimately depended on students’ voluntary consent at the time of data collection.

The final sample consisted of 298 Social Work students from nine Chilean universities, both public and private. To ensure data completeness, the mandatory response option was activated in QuestionPro, preventing missing values.

Although sociodemographic information such as gender, ethnicity, and urban/rural residence was collected for the purpose of characterizing the participant sample (Table 1), these variables were not incorporated into the inferential analyses of the present article. This decision was made because the primary aim of the study was specifically focused on evaluating the structural relationships between loneliness, academic organization, and academic stress among Social Work students. Therefore, the analysis of potential differences or effects associated with sociodemographic variables fell outside the analytical scope of this research.

### 2.2. Instruments

Firstly, a sociodemographic questionnaire was applied, which collected identifying information about the students. The instrument included closed-ended questions such as the following: gender, age, belonging to an ethnic group, university, and place of residence.

Additionally, the Spanish version of the Freshman Stress Questionnaire (FSQ) was used, adapted by García-Guerrero (2011) from the instrument originally developed by Boujut and Bruchon-Schweitzer (2009). Unlike the original version, the García-Guerrero (2011) adaptation comprises 17 items distributed across four dimensions: academic stress (6 items), university dysfunctions (4 items), loneliness (4 items), and problems with close relations (3 items). Responses are rated on a five-point ordinal scale (1 = not at all, 5 = very much). In the present study, only the loneliness and academic stress dimensions were used. Both dimensions demonstrated adequate internal consistency indices (loneliness: ω = 0.779; α = 0.776; academic stress: ω = 0.756; α = 0.755) (González-Casas et al., 2022).

Lastly, the Academic Performance Scale (Preciado-Serrano et al., 2021) was applied. The original instrument is composed of five dimensions related to academic functioning: contribution to academic activities (10 items), dedication to study (5 items), lack of academic organization (5 items), grade point average (3 items), and quality of study (3 items). In the present study, only the lack of academic organization dimension was used. The subscale demonstrated adequate internal consistency indices (ω = 0.765; α = 0.757).

### 2.3. Procedure

For the implementation of the study, institutional authorization was obtained from the academic authorities of the participating universities, ensuring formal access to the student population and compliance with institutional regulations related to research. The study was approved by the Scientific Ethics Committee of Universidad de La Frontera (UFRO File No. 064-24), in accordance with national and international ethical principles governing research involving human participants. This approval ensured compliance with criteria related to voluntary participation, informed consent, confidentiality, anonymity, and the protection of personal data throughout all stages of the research process.

Data collection was carried out between September and December 2024 through an online questionnaire administered via the QuestionPro platform. Students were invited to participate through emails sent by the research team, which included a general description of the study, the informed consent form, and a direct link to the questionnaire. Before accessing the instrument, participants were required to review and electronically accept the informed consent form, which detailed the objectives of the research, the voluntary nature of participation, conditions of confidentiality and anonymity, the absence of foreseeable risks associated with the study, and the right to withdraw at any time without academic or personal consequences.

In addition, the collaboration of three expert judges in Chile was sought to evaluate the FSQ and Academic Performance Scale instruments. The review focused on the semantic relevance, conceptual clarity, and cultural appropriateness of the items within the Chilean university context. The specialists’ evaluation indicated that the items were comprehensible, relevant, and suitable for the target population, thereby contributing to strengthening the content validity and contextual relevance of the instruments used in the study.

### 2.4. Data Analysis

To achieve the research objectives, descriptive and correlational analyses were initially conducted using the SPSS software version 25, which allowed us to characterize the variables and explore their preliminary associations. Subsequently, structural equation models (SEMs) were estimated using Mplus version 8.11 (Muthén & Muthén, 2017), with the aim of comprehensively examining the relationships among the proposed constructs.

The Weighted Least Squares Mean and Variance adjusted (WLSMV) method was used to estimate the parameters; this method is widely recommended when working with variables of ordinal nature due to its robustness against deviations from normality and its adequate precision with this type of data (Bagheri & Saadati, 2021).

The structural model included three factors: loneliness, academic stress, and academic organization, specifying direct relationships among them. Likewise, the presence of a mediating effect was assessed using the MODEL INDIRECT command in Mplus, which allows for the simultaneous estimation of total, direct, and indirect effects, along with their respective levels of statistical significance (*p*-values) and 95% confidence intervals.

Model fit was examined using various goodness-of-fit indices. In particular, the Comparative Fit Index (CFI) and the Tucker–Lewis Index (TLI) were considered, with values of 0.90 or higher taken as indicative of acceptable fit (Schumacker & Lomax, 2016). Complementarily, the Root Mean Square Error of Approximation (RMSEA) and the Standardized Root Mean Square Residual (SRMR) were analyzed, for which values equal to or less than 0.08 were adopted as criteria, in accordance with widely validated recommendations in the methodological literature (Dueber, 2017; Gouveia et al., 2018).

Finally, to interpret the magnitude of the structural effects, the cutoff points proposed by Miranda-Zapata et al. (2018) were followed, classifying the effects as low (<0.30), moderate (between 0.30 and 0.50), and high (>0.50).

## 3. Results

### 3.1. Descriptive Analysis

Table 2 presents Pearson’s r correlations between the study variables, along with their descriptive statistics. The results show positive and statistically significant associations between the factors. In particular, the lack of Academic Organization (t) exhibits a low-to-moderate correlation with both Academic Stress (y) and Loneliness (x). In turn, Academic Stress is moderately and positively related to Loneliness. The lower part of the table includes the descriptive statistics for each variable means, standard deviations, skewness, and kurtosis, which allow for a more detailed characterization of their behavior in the sample. These results support the use of robust estimators, consistent with the ordinal nature of the scales.

### 3.2. Evaluation of the Structural Mediation Model

**Figure 2:**
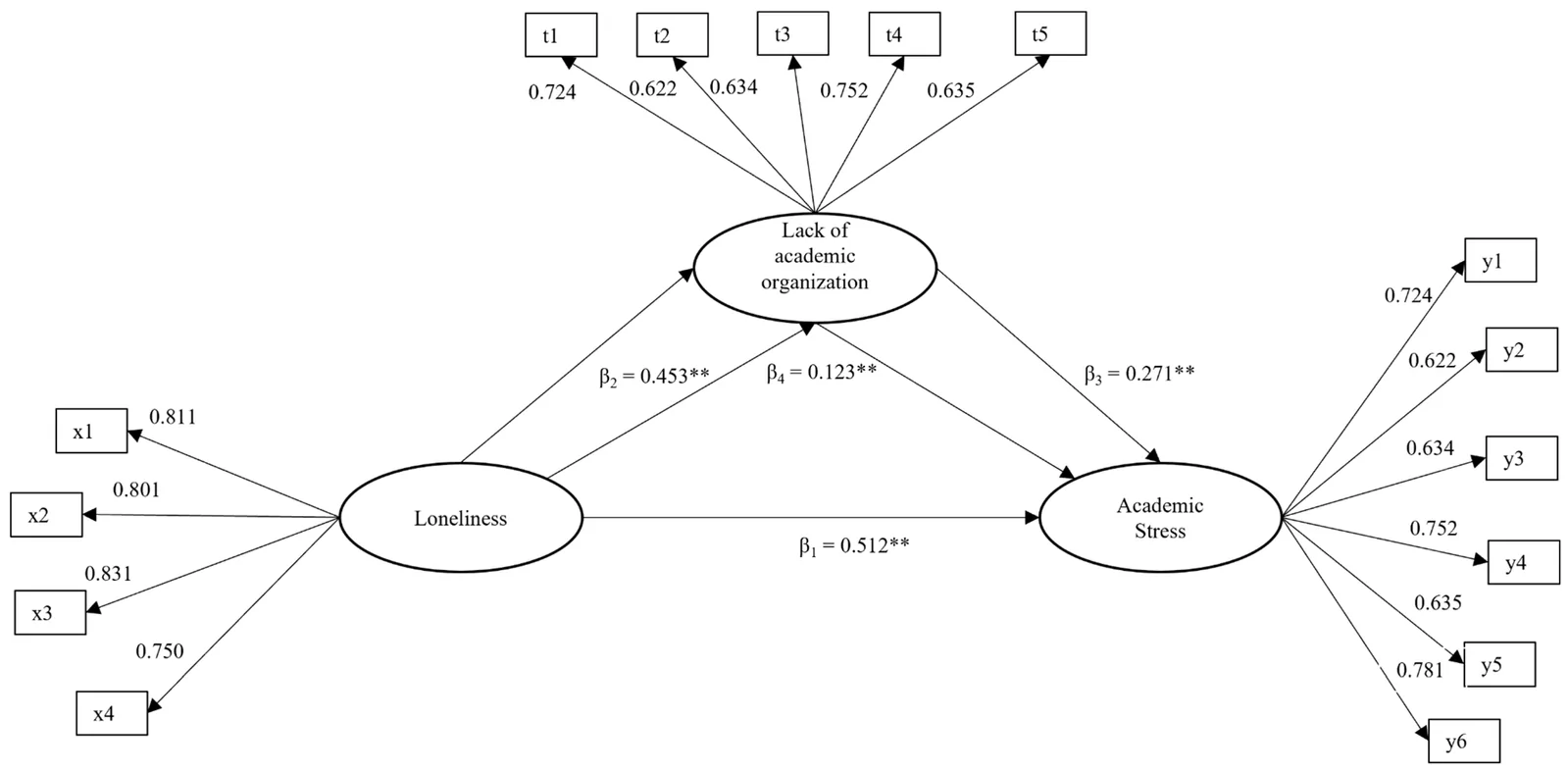
Structural model with standardized coefficients. Note. Rectangles represent observed variables (items), and ellipses represent latent constructs. Values shown correspond to standardized factor loadings and standardized structural path coefficients (STDYX). Loneliness is specified as the independent variable, lack of academic organization as the mediator, and academic stress as the outcome variable. β_1_, β_2_, and β_3_ represent direct effects, whereas β_4_ represents the standardized indirect effect associated with the mediation pathway. All structural paths shown in the model are statistically significant. ** indicates *p* < 0.01.

Figure 2 presents the main results of the proposed structural model. The goodness-of-fit indices indicate that the model showed an adequate overall fit to the data, with a significant value for the χ^2^ test of WLSMV (χ^2^ = 277.829, df = 85, *p* < 0.001), which is to be expected given the sensitivity of this statistic to sample size. However, the incremental indices reached satisfactory values (CFI = 0.937; TLI = 0.922), while the fit and absolute parsimony indices fell within acceptable ranges (SRMR = 0.055; RMSEA = 0.078). These results suggest that the hypothesized mediation model provides a theoretically coherent and empirically supported representation of the relationships between the study variables.

Regarding the results of the mediation model, all four hypotheses were confirmed. Regarding Hypothesis 1, loneliness had a direct, positive, strong, and statistically significant effect on academic stress (β_1_ = 0.512, *p* < 0.01), indicating that higher levels of loneliness are associated with higher levels of academic stress. Regarding Hypothesis 2, loneliness had a direct, positive, moderate, and statistically significant effect on lack of academic organization (β_2_ = 0.453, *p* < 0.01). Regarding Hypothesis 3, lack of academic organization showed a direct, positive, and statistically significant effect on academic stress (β_3_ = 0.271, *p* < 0.01), indicating that high levels of lack of academic organization are associated with higher levels of academic stress.

Regarding Hypothesis 4, the results indicated that the relationship between loneliness and academic stress is significantly mediated by a lack of academic organization. Specifically, a positive, small, and statistically significant standardized indirect effect was observed (β_4_ = 0.123, *p* < 0.01). Overall, the total standardized effect of loneliness on academic stress was positive, high, and statistically significant (β_total_ = 0.635, *p* < 0.01), reflecting the combination of direct and indirect effects. Since the direct effect remained statistically significant, the results indicate the presence of partial mediation. Taken together, these findings suggest that loneliness increases academic stress both directly and indirectly, through greater difficulties in academic organization.

## 4. Discussion

The findings of the present study suggest that loneliness constitutes more than an individual emotional experience among Social Work students, as it also reflects broader difficulties in sustaining meaningful support networks within demanding university environments. In this sense, the association between loneliness and academic stress may be interpreted as an expression of the tensions produced by highly individualized educational trajectories, where students are expected to respond autonomously to increasing academic and emotional demands. Within Social Work education, this issue acquires relevance because students are frequently exposed to emotionally demanding professional training contexts, internships, and institutional pressures that can intensify feelings of vulnerability and exhaustion (Baird, 2016; Pham et al., 2023). Consequently, loneliness may weaken not only emotional well-being but also students’ perceived capacity to cope effectively with academic responsibilities.

The results also indicate that difficulties in academic organization play an important role in students’ experiences of stress. Rather than being understood exclusively as an individual deficit in time management, academic disorganization may reflect structural tensions within higher education environments characterized by excessive workloads, overlapping assessments, and limited institutional support. From this perspective, stress emerges not only from the volume of academic demands, but also from the perception that students lack sufficient resources and organizational conditions to respond adequately to them. This interpretation is especially relevant in Social Work programs, where academic responsibilities coexist with emotionally intensive training experiences that require sustained levels of self-regulation and adaptation.

One of the most relevant findings of this study is that lack of academic organization partially mediates the relationship between loneliness and academic stress. This suggests that loneliness may indirectly intensify stress by affecting students’ ability to structure study routines, anticipate academic demands, and maintain stable learning processes. In this regard, the findings support the notion that psychosocial well-being and academic functioning are deeply interconnected dimensions within the university experience. The weakening of social bonds may reduce opportunities for collaborative learning, emotional validation, and peer support, thereby increasing students’ vulnerability to disorganization and overload. Thus, the relationship observed in this study should not be interpreted solely at the individual level, but also as a reflection of institutional and relational conditions that shape the educational experience in higher education.

These findings are particularly significant for Social Work education because the profession itself is grounded in relational processes, emotional support, and human interaction. Experiencing loneliness and sustained academic stress during professional training may therefore have implications not only for students’ well-being, but also for the development of professional competencies associated with empathy, intervention, and psychosocial support. In this context, the results reinforce the importance of promoting educational environments that strengthen social connectedness, peer support, and institutional accompaniment throughout the university trajectory. This becomes especially relevant in careers such as Social Work, where academic formation is strongly linked to emotional demands and exposure to complex social realities.

From an applied perspective, the findings highlight the need to move beyond individual-centered approaches to academic stress and toward more comprehensive institutional strategies. Interventions aimed solely at improving personal coping skills may be insufficient if university environments continue reproducing conditions associated with overload, fragmentation, and limited support. Consequently, higher education institutions should strengthen mechanisms for academic accompaniment, psychosocial support, and student integration, while also promoting organizational conditions that facilitate healthier and more sustainable educational trajectories. In the context of Social Work education, these strategies may contribute not only to reducing academic stress, but also to fostering more inclusive and supportive professional training environments.

Finally, this study contributes to the literature by integrating loneliness, lack of academic organization, and academic stress into a structural equation model, allowing for a more comprehensive understanding of their direct and indirect relationships. Beyond confirming statistical associations, the findings provide evidence that academic stress among Social Work students should be understood as a multidimensional phenomenon shaped by relational, organizational, and institutional factors. From this perspective, strengthening student well-being requires not only individual interventions, but also institutional transformations capable of promoting social integration, academic support, and healthier educational environments within higher education.

### Limitations and Future Research

Despite the study’s significant findings and its theoretical and practical implications, certain limitations must be acknowledged. First, the cross-sectional design prevents the establishment of causal relationships between the variables under examination. Although the structural model enables the estimation of direct and indirect associations consistent with the previous literature (Gálvez-Nieto et al., 2025), it is not possible to determine the temporal directionality of the observed effects. Consequently, the identified relationships should be interpreted as associations rather than causal relationships.

One additional limitation relates to the assessment of loneliness. In the present study, loneliness was measured using a subscale embedded within the Freshman Stress Questionnaire rather than through a specific loneliness instrument such as the UCLA Loneliness Scale or the De Jong Gierveld Loneliness Scale. Consequently, loneliness was assessed from the perspective of stress-related university adjustment, which may not fully capture the multidimensional nature of the construct. Therefore, the findings should be interpreted cautiously, as they reflect a specific dimension of loneliness associated with academic stress experiences rather than a comprehensive assessment of loneliness.

One limitation of the present study relates to the gender composition of the sample, which is characterized by a higher presence of women, a situation that is frequent in university programs linked to the field of Social Work. This distribution could influence the configuration and the relationship between the analyzed variables; therefore, the results must be interpreted given this demographic skew (Gálvez-Nieto et al., 2025). Previous research has indicated that academic experiences, as well as levels of stress and student well-being, may vary according to gender (Ball et al., 2024).

A further limitation is that, although sociodemographic information such as gender, ethnicity, and urban/rural residence was collected to characterize the sample, these variables were not incorporated into the analyses presented in this article. In this regard, future research could further examine the potential influence of these characteristics on the relationships observed among the study variables, identifying relevant differences between groups and contributing to a more contextualized understanding of the phenomena analyzed.

As future lines of research, we recommend developing longitudinal studies to analyze how loneliness, academic organization, and academic stress evolve over the course of a student’s university trajectory. Such designs would allow for a deeper understanding of the temporal dynamic of these variables, as well as the identification of critical moments in the educational process during which strategies of academic support and student well-being could be implemented (Baelen et al., 2026; Klenner-Loebel et al., 2026).

Future studies could also incorporate specific and multidimensional instruments for the assessment of loneliness in university populations to broaden the understanding of this construct and compare the consistency of findings between different measurement approaches. Likewise, future research could incorporate more balanced sampling strategies to more precisely examine potential gender differences in the relationship between loneliness, academic organization, and academic stress.

In broader terms, the present findings contribute to the understanding of academic stress among Social Work students within the Chilean higher education context. Future research could continue exploring the role of variables such as perceived social support, academic resilience, self-efficacy, coping strategies, and institutional belonging, which may be relevant to students’ academic well-being and university adjustment. Likewise, additional studies conducted in other institutional and cultural contexts would help broaden the interpretation and transferability of the findings.

## 5. Conclusions

The results of this study provide relevant empirical evidence on the role of loneliness, lack of organization, and academic stress in the university experience of Social Work students. Taken together, the findings show that these dimensions are interrelated and configure a psychosocial framework that influences students’ academic well-being. In particular, higher levels of disorganization regarding university tasks and demands are associated with an increase in academic stress, while loneliness emerges as a factor that deepens the tensions experienced during professional training. These results reinforce the need to understand university life not only in terms of academic performance but also in terms of the relational and organizational conditions that influence the educational experience of future social workers.

From a disciplinary perspective, this study makes a substantial contribution to the field of Social Work by highlighting how certain psychosocial processes present in university life influence the training of those who will later work in complex social contexts. Analyzing loneliness, academic organization, and stress among students in this discipline allows us to broaden the understanding of student well-being from a perspective grounded in professional training. In this regard, the study provides evidence that can enrich the academic discussion on the factors influencing the educational trajectory of Social Work students, thereby strengthening research that integrates personal, academic, and contextual dimensions into the analysis of university well-being.

In practical terms, the study findings suggest the need to strengthen institutional strategies aimed at promoting student well-being within Social Work education, particularly through academic support programs, the promotion of self-care, and the strengthening of peer support networks among students. Likewise, it is important to implement preventive actions that foster academic organization and mental health, thereby contributing to more sustainable and inclusive educational trajectories in higher education.

## Figures and Tables

**Table 1:** Sociodemographic characterization of the sample.

Variable	Categories	*n* (%)
Sex	Men	23.1%
	Women	76.9%
Ethnic group	No	78.6%
	Yes	21.4%
Residence	Urban	76%
	Rural	24%

**Table 2:** Pearson’s r correlation matrix and descriptive statistics.

**Factors**	**t**	**y**	**x**
t (Lack of organization)	1	0.371 **	0.346 **
y (Academic Stress)	0.371 **	1	0.516 **
x (Loneliness)	0.346 **	0.516 **	1
**Descriptive Statistics**	**t**	**y**	**x**
Mean	15.000	20.590	9.717
SD	5.489	5.230	4.486
g1	1.381	−0.216	0.648
g2	2.699	−0.607	−0.499

Note: The coefficients correspond to Pearson’s r correlations. SD = standard deviation; g1 = skewness; g2 = kurtosis. ** *p* < 0.001. t = Lack of academic organization; y = Academic stress; x = Loneliness.

## Data Availability

The data presented in this study are available on request from the corresponding author due to ethical restrictions.

## References

[B1-behavsci-16-01439] Afrouz R., Crisp B. R. (2021). Online education in social work, effectiveness, benefits, and challenges: A scoping review. Australian Social Work.

[B2-behavsci-16-01439] Akyürek M. İ. (2021). Üniversite öğrencilerinin zaman yönetimi becerileri. Yükseköğretim Dergisi.

[B3-behavsci-16-01439] Alinejad V., Parizad N., Yarmohammadi M., Radfar M. (2022). Loneliness and academic performance mediates the relationship between fear of missing out and smartphone addiction among Iranian university students. BMC Psychiatry.

[B4-behavsci-16-01439] American Psychiatric Association (2023). Fostering college student mental health and resilience.

[B5-behavsci-16-01439] Aslan Y., Koçak O., Kaya A. B., Alkhulayfi A. M. A., Gómez-Salgado J., Yıldırım M. (2025). Roles of loneliness and life satisfaction in the relationship between perceived friend social support, positive feelings about the future and loss of motivation. Acta Psychologica.

[B6-behavsci-16-01439] Baelen R. N., Lovett J. M., Thursby Bourke K., Galloway C., Parker A., Baghdady A., Schonert-Reichl K. A. (2026). Leveraging evidence on relations between teacher well-being and student well-being and learning: A scoping review. Review of Educational Research.

[B7-behavsci-16-01439] Bagheri A., Saadati M. (2021). Generalized structural equations approach in the of elderly self-rated health. Journal of Physics: Conference Series.

[B8-behavsci-16-01439] Baird S. L. (2016). Conceptualizing anxiety among social work students: Implications for social work education. Social Work Education.

[B9-behavsci-16-01439] Ball I., Banerjee M., Holliman A., Tyndall I. (2024). Investigating success in the transition to university: A systematic review of personal risk and protective factors influencing academic achievement. Educational Psychology Review.

[B10-behavsci-16-01439] Ball I., Banerjee M., Holliman A., Tyndall I. (2025). Investigating success in the transition to university: A systematic review of personal risk and protective factors influencing psychosocial success. Educational Psychology Review.

[B11-behavsci-16-01439] Bauman Z. (2003). Amor líquido: Acerca de la fragilidad de los vínculos humanos.

[B12-behavsci-16-01439] Beck U., Beck-Gernsheim E. (2002). Individualization: Institutionalized individualism and its social and political consequences.

[B13-behavsci-16-01439] Behera N., Khuntia S., Pandey K., Shankar S. (2025). Impact of social media use on physical, mental, social, and emotional health, sleep quality, body image, and mood: Evidence from 21 countries—A systematic literature review with narrative synthesis. International Journal of Behavioral Medicine.

[B14-behavsci-16-01439] Benítez-Agudelo J. C., Restrepo D., Navarro-Jimenez E., Clemente-Suárez V. J. (2025). Longitudinal effects of stress in an academic context on psychological well-being, physiological markers, health behaviors, and academic performance in university students. BMC Psychology.

[B15-behavsci-16-01439] Boladeres T. S., Rodríguez J. A. L., Ramajo B. P. (2024). Las competencias educativas para el aprendizaje de la identidad profesional del Trabajo social en las universidades españolas. Interacción y Perspectiva: Revista de Trabajo Social.

[B16-behavsci-16-01439] Boujut E., Bruchon-Schweitzer M. (2009). A construction and validation of a freshman stress questionnaire: An exploratory study. Psychological Reports.

[B17-behavsci-16-01439] Boynton H. M. (2025). Establishing spirituality competencies in social work. Journal of Religion & Spirituality in Social Work: Social Thought.

[B18-behavsci-16-01439] Brambila-Tapia A. J. L., Velarde-Partida E. U., Carrillo-Delgadillo L. A., Macías-Espinoza F., Ramírez-De los Santos S. (2025). Association between academic, cognitive and health-related variables with academic stress in health sciences university students. Behavioral Sciences.

[B19-behavsci-16-01439] Brown E. G., Gallagher S., Creaven A. M. (2018). Loneliness and acute stress reactivity: A systematic review of psychophysiological studies. Psychophysiology.

[B20-behavsci-16-01439] Brunsting N. C., Zachry C., Liu J., Bryant R., Fang X., Wu S., Luo Z. (2021). Sources of perceived social support, social-emotional experiences, and psychological well-being of international students. The Journal of Experimental Education.

[B21-behavsci-16-01439] Calle-Müller C., ElZomor M. (2025). Prioritizing mental health to improve academic performance and enhance construction engineering and management students’ well-being. Journal of Civil Engineering Education.

[B22-behavsci-16-01439] Campagne D. M. (2019). Stress and perceived social isolation (loneliness). Archives of Gerontology and Geriatrics.

[B23-behavsci-16-01439] Chust-Hernández P., López-González E., Senent-Sánchez J. M. (2023). Efficacy of non-pharmacological interventions to reduce academic stress in higher education students: A systematic review. Ansiedad y Estrés.

[B24-behavsci-16-01439] Chust-Hernández P., López-González E., Senent-Sánchez J. M. (2024). Study skills intervention addressing academic stress and self-efficacy in newly admitted university students. Electronic Journal of Research in Educational Psychology.

[B25-behavsci-16-01439] Dominguez-Lara S., Valente S. N., Lourenço A. A., Peceros-Pinto B., Díaz-Peñaloza M., León S. R. (2024). Apoyo social percibido y rendimiento académico en estudiantes de secundaria peruanos: Influencia del engagement académico y de la autoeficacia académica. Revista Fuentes.

[B26-behavsci-16-01439] Dueber D. M. (2017). Bifactor indices calculator: A Microsoft Excel-based tool to calculate various indices relevant to bifactor CFA models.

[B27-behavsci-16-01439] Ellard O. B., Dennison C., Tuomainen H. (2023). Interventions addressing loneliness amongst university students: A systematic review. Child and Adolescent Mental Health.

[B28-behavsci-16-01439] Estrada-Araoz E. G., Ayay-Arista G., Cruz-Laricano E. O., Paricahua-Peralta J. N. (2024). Estresores académicos y los estilos de vida de los estudiantes universitarios: Un estudio predictivo en una universidad pública [Academic stressors and the lifestyles of university students: A predictive study at a public university]. Retos.

[B29-behavsci-16-01439] Gao X. (2023). Academic stress and academic burnout in adolescents: A moderated mediating model. Frontiers in Psychology.

[B30-behavsci-16-01439] García-Guerrero A. M. (2011). Efectos del estrés percibido y las estrategias de aprendizaje cognitivas en el rendimiento académico de estudiantes universitarios noveles de ciencias de la salud.

[B31-behavsci-16-01439] Gálvez-Nieto J. L., Davinson-Pacheco G., Tereucán-Angulo J., Norambuena-Paredes I., Briceño-Olivera C., Briceño-Olivera X., Rodríguez-Martín V. (2025). Exploring the relationships between academic engagement and professional suitability in social work students: The mediating role of academic satisfaction. Behavioral Sciences.

[B32-behavsci-16-01439] González-Casas D., Barbé A. I. D., Mercado García E., Calleja Jimenez J. P., Gálvez-Nieto J. L. (2022). Psychometric examination of the freshman stress questionnaire using a sample of social work students in Spain during the COVID-19 pandemic. The British Journal of Social Work.

[B33-behavsci-16-01439] Gouveia V. V., de Moura H. M., Santos L. C. D. O., do Nascimento A. M., Guedes I. D. O., Gouveia R. S. V. (2018). Escala de autorrelato de trapaça-admissão: Evidências de validade fatorial e precisão. Revista Colombiana de Psicología.

[B34-behavsci-16-01439] Gutierrez A. B. B., Blanco E., Herrero E. T., Diez F. J. H. (2025). Academic stress and socio-demographic variables as predictors of university dropout intention. Electronic Journal of Research in Education Psychology.

[B35-behavsci-16-01439] Hadad S. (2025). Learning in crisis: Self-regulation, routine-facilitated well-being, and academic stress among higher education students. Metacognition and Learning.

[B36-behavsci-16-01439] Hamdan-Mansour A. M., Bani-Hani M. A., Alshibi A. N., Hamdan-Mansour R. A., Mahmoud A. K., Hussein A. K., Marmash L., Al Shuaibi J., Mansour L. A. H. (2025). The moderating effect of academic stress on the relationship between self-esteem, loneliness, personality traits, academic procrastination, and suicidality among university students in Jordan. Health Psychology Research.

[B37-behavsci-16-01439] Hamdan-Mansour A. M., Hamdan-Mansour R. A., Allaham D. M., Alrashidi M., Alhaiti A., Mansour L. A. H. (2024). Academic procrastination, loneliness, and academic anxiety as predictors of suicidality among university students. International Journal of Mental Health Nursing.

[B38-behavsci-16-01439] Han B.-C. (2014). La agonía del eros.

[B39-behavsci-16-01439] Harris B. R., Maher B. M., Wentworth L. (2022). Optimizing efforts to promote mental health on college and university campuses: Recommendations to facilitate usage of services, resources, and supports. The Journal of Behavioral Health Services & Research.

[B40-behavsci-16-01439] Hussin S. H., Awang Daud A. I., Taibi M., Hussin S. R. (2021). Loneliness, coping strategies and perceived social support among students of public universities in Malaysia during the COVID-19 MCO. International Journal of Business & Society.

[B41-behavsci-16-01439] Inquilla-Mamani J., López-Cueva M. A., Mamani E. F. (2025). Procrastinación académica y fracaso académico de los estudiantes universitarios en una universidad pública peruana. Revista Pedagogía Universitaria y Didáctica del Derecho.

[B42-behavsci-16-01439] Islam M. S., Rabbi M. F. (2024). Exploring the sources of academic stress and adopted coping mechanisms among university students. International Journal on Studies in Education.

[B43-behavsci-16-01439] Iwasaki S., Deguchi Y., Inoue K. (2025). Loneliness and psychological stress in Japanese university students: A cross-sectional gender-based comparative study. Psychiatry and Clinical Neurosciences Reports.

[B44-behavsci-16-01439] Jefferson R., Barreto M., Jones F., Conway J., Chohan A., Madsen K. R., Verity L., Petersen K. J., Qualter P. (2023). Adolescent loneliness across the world and its relation to school climate, national culture and academic performance. British Journal of Educational Psychology.

[B45-behavsci-16-01439] Jiang L., Fang L., Xu Y., Zhang Q., Dai S., Tian J., Wu W., Fang Y., Zhang M., Yu H. (2025). How does emotional exhaustion among Chinese college students affect mental health? A mixed-methods study in Zhejiang, China. Frontiers in Public Health.

[B46-behavsci-16-01439] Kangasniemi M., Karki S., Voutilainen A., Saarnio R., Viinamäki L., Häggman-Laitila A. (2022). The value that social workers’ competencies add to health care: An integrative review. Health & Social Care in the Community.

[B47-behavsci-16-01439] Klenner-Loebel M., Gálvez-Nieto J. L., Véliz J. C. B., Astudillo C. G. O., Norambuena-Paredes I. (2026). Psychometric properties of the intercultural effectiveness scale in a sample of in-service Chilean EFL teachers. Frontiers in Psychology.

[B48-behavsci-16-01439] Klinkenberg E. F., Versteeg M., Kappe R. F. (2024). Engagement and emotional exhaustion among higher education students; a mixed-methods study of four student profiles. Studies in Higher Education.

[B49-behavsci-16-01439] Korzhina Y., Hemberg J., Nyman-Kurkiala P., Fagerström L. (2022). Causes of involuntary loneliness among adolescents and young adults: An integrative review. International Journal of Adolescence and Youth.

[B50-behavsci-16-01439] Kulari G., Ribeiro L., Tomé Pires C. (2026). Friendship matters: Indirect effect of social support from friends on depressive symptoms and loneliness among university students. Studies in Higher Education.

[B51-behavsci-16-01439] Lei W., Wang X., Dai D. Y., Guo X., Xiang S., Hu W. (2022). Academic self-efficacy and academic performance among high school students: A moderated mediation model of academic buoyancy and social support. Psychology in the Schools.

[B52-behavsci-16-01439] Leich M., Guse J., Bergelt C. (2025). Loneliness and mental burden among German medical students during the fading COVID-19 pandemic: A mixed-methods approach. Frontiers in Psychology.

[B53-behavsci-16-01439] Li R., Fu W., Liang Y., Huang S., Xu M., Tu R. (2024). Exploring the relationship between resilience and internet addiction in Chinese college students: The mediating roles of life satisfaction and loneliness. Acta Psychologica.

[B54-behavsci-16-01439] Lin Y., Fan Z. (2023). The relationship between rejection sensitivity and social anxiety among Chinese college students: The mediating roles of loneliness and self-esteem. Current Psychology.

[B55-behavsci-16-01439] Liu Y., Li Z. (2023). International research review and teaching improvement measures of college students’ learning psychology under the background of COVID-19. Sustainability.

[B56-behavsci-16-01439] López-García A., Blasco-Blasco O., Liern-García M., Parada-Rico S. E. (2023). Early detection of students’ failure using Machine Learning techniques. Operations Research Perspectives.

[B57-behavsci-16-01439] Malakcioglu C. (2024). Emotional loneliness, perceived stress, and academic burnout of medical students after the COVID-19 pandemic. Frontiers in Psychology.

[B58-behavsci-16-01439] Melaku B. K., Darge Negasi R., Tamiru Tolla T. (2025). Academic resilience mediating perceived academic support and achievement in first-year university students. Cogent Education.

[B59-behavsci-16-01439] Miranda-Zapata E., Lara L., Navarro J. J., Saracostti M., de-Toro X. (2018). Modelling the effect of school engagement on attendance to classes and school performance. Revista de Psicodidáctica (English Ed.).

[B60-behavsci-16-01439] Mojibpour M., Salehi A., Shadzi M. R., Derakhshani S. (2025). Exploring associations between problematic technology-dependent behaviors, physical and mental health, sleep quality, and academic procrastination among medical students. BMC Public Health.

[B61-behavsci-16-01439] Moriña A., Biagiotti G. (2022). Academic success factors in university students with disabilities: A systematic review. European Journal of Special Needs Education.

[B62-behavsci-16-01439] Motloung S., Mzinyane B. (2023). Adapting the social work curriculum for relevance in a South African university: A collaborative autoethnography of social work academics during the KZN floods. Social Work/Maatskaplike Werk.

[B63-behavsci-16-01439] Muthén L. K., Muthén B. (2017). Mplus user’s guide: Statistical analysis with latent variables, user’s guide.

[B64-behavsci-16-01439] Niazov Z., Hen M., Ferrari J. R. (2022). Online and academic procrastination in students with learning disabilities: The impact of academic stress and self-efficacy. Psychological Reports.

[B65-behavsci-16-01439] Norambuena-Paredes I. (2025). La comprensión de sí y el encuentro con el otro: Reflexiones sobre educación y alteridad en la sociedad contemporánea. Desde el Sur.

[B66-behavsci-16-01439] Norambuena-Paredes I., Polanco-Levicán K., Tereucán-Angulo J., Sepúlveda-Maldonado J., Gálvez-Nieto J. L., Tavera-Cuellar C., Pérez-Ramírez S., Álvarez-Violante C., López-Tarango R. (2025). Factorial invariance of the satisfaction with life scale (SWLS) in Mexican and Colombian university students. Behavioral Sciences.

[B67-behavsci-16-01439] Norazizi N. D. K., Amat M. A. C., Mohammad Hanafi N. H., Salam S. N. (2024). Influence of depression and loneliness on suicidal behaviour among public university students in Malaysia. Pertanika Journal of Social Sciences & Humanities.

[B68-behavsci-16-01439] Ostrow K. D., Rieur O., Moeller R. W., Seehuus M. (2025). From sleeplessness to solitude: Emotional repair as a buffer between insomnia and loneliness in university students. Frontiers in Sleep.

[B69-behavsci-16-01439] Özdoğan A. Ç. (2021). The mediating role of loneliness in the relationship between parental emotional availability, romantic relationship quality, and social connectedness and well-being. Pamukkale Universitesi Egitim Fakultesi Dergisi/Pamukkale University Journal of Education.

[B70-behavsci-16-01439] Patzak A., Zhang X., Vytasek J. (2025). Boosting productivity and wellbeing through time management: Evidence-based strategies for higher education and workforce development. Frontiers in Education.

[B71-behavsci-16-01439] Peker M. (2024). Willing, able, and engaged: Roles of action-state orientation, intrinsic academic motivation, and time management on academic engagement. Current Psychology.

[B72-behavsci-16-01439] Peris-Ramos H. C., David-Fernandez S., Beltrán-Velasco A. I., Yáñez-Sepúlveda R., López-Gil J. F., Clemente-Suárez V. J. (2025). Stress and wellness paradigms among educators in diverse academic contexts in Spain, Colombia and Chile. Frontiers in Education.

[B73-behavsci-16-01439] Pham N., Hung N., Tran S. G., Dung N., Le V., Tran T. K. H., Nguyen K. O., Vu T. T. M., Nguyen T. P., Xuan L. N., Liem N. (2023). Hospital social work education in Vietnam: Achievements, challenges, and lessons learned. Social Work Education.

[B74-behavsci-16-01439] Potter J., Chatterjee A., Bristow J., Cant S. (2025). Making up for lost time: University students’ quest to reclaim missed opportunities while adjusting to post-Covid life in higher education. British Educational Research Journal.

[B75-behavsci-16-01439] Preciado-Serrano M., Ángel-González M., Colunga-Rodríguez C., Vázquez-Colunga J. C., Esparza-Zamora M. A., Vázquez-Juárez C. L., Obando-Changuán M. P. (2021). Construcción y validación de la Escala RAU de rendimiento académico universitario. Revista Iberoamericana de Diagnóstico y Evaluación-e Avaliação Psicológica.

[B76-behavsci-16-01439] Qian L., Fuqiang Z. (2018). Academic stress, academic procrastination and academic performance: A moderated dual-mediation model. Journal on Innovation and Sustainability RISUS.

[B77-behavsci-16-01439] Quan L., Zhen R., Yao B., Zhou X. (2014). The effects of loneliness and coping style on academic adjustment among college freshmen. Social Behavior and Personality: An International Journal.

[B78-behavsci-16-01439] Real-Delor R., Guevara Tirado A., Morales Ojeda I., Chibas Muñóz E., Cañete Cáceres E., Carballo Almeida M., Flor Lugo D., Noldin Villasanti A., Pereira Salles V., Sanabria Cañete N., Alvarenga Ferreira M., Ríos Pagnussatt M., Zaracho Miranda A., González Vera G. (2024). Factores asociados al rendimiento académico en estudiantes universitarios de Latinoamérica en 2023. Investigación En Educación Médica.

[B79-behavsci-16-01439] Romero-Pérez C., Sánchez-Lissen E. (2022). Scientific narratives in the study of student time management: A critical review. International and Multidisciplinary Journal of Social Sciences.

[B80-behavsci-16-01439] Salari N., Zarei H., Rasoulpoor S., Ghasemi H., Hosseinian-Far A., Mohammadi M. (2025). The impact of social networking addiction on the academic achievement of university students globally: A meta-analysis. Public Health in Practice.

[B81-behavsci-16-01439] Salguero-Pazos M. R., Reyes-de-Cózar S. (2023). Interventions to reduce academic procrastination: A systematic review. International Journal of Educational Research.

[B82-behavsci-16-01439] San C. K., Guo H. (2023). Institutional support, social support, and academic performance: Mediating role of academic adaptation. European Journal of Psychology of Education.

[B83-behavsci-16-01439] Scheaffer R. L., Mendenhall W., Ott L., Rendón G., Gómez J. R., Vargas S. (2000). Elementos de Muestreo.

[B84-behavsci-16-01439] Schumacker R., Lomax R. G. (2016). A beginner’s guide to structural equation modelling.

[B85-behavsci-16-01439] Sedláková E., Křeménková L. (2019). Social support among university students. EDULEARN19 proceedings.

[B86-behavsci-16-01439] Sing-Yun W. (2023). Digitalization challenges in education during COVID-19: A systematic review. Cogent Education.

[B87-behavsci-16-01439] Södel C. A., Herr R. M., Deyerl V. M., Diehl K. (2025). Individual determinants of perceived university injustice and its association with loneliness and well-being: A cross-sectional study from Germany. Journal of Public Health.

[B88-behavsci-16-01439] Stasolla F., Zullo A., Maniglio R., Passaro A., Di Gioia M., Curcio E., Martini E. (2025). Deep learning and reinforcement learning for assessing and enhancing academic performance in university students: A scoping review. AI.

[B89-behavsci-16-01439] Stead J., Gracia L., Riva E. (2025). An investigation into the wellbeing and loneliness of PGR students and the effectiveness of institutional support strategies. European Journal of Education.

[B90-behavsci-16-01439] Tadesse T., Fischer M. R., Ataro G., Gedamu S., Jebessa M., Mamaru A., Siebeck M. (2024). Prevalence of anxiety and depression symptoms among university teachers in Ethiopia during the COVID-19 pandemic. Healthcare.

[B91-behavsci-16-01439] Troncone A., Caldarelli G., Cosenza M., Affuso G., Sacco M., Ciccarelli M., Pizzini B. (2025). Psychological problems and academic motivation in university students: A cross-sectional study one year after the COVID-19 lockdown in Italy. Psychiatric Quarterly.

[B92-behavsci-16-01439] Ueno A., Yu C., Curtis L., Dennis C. (2025). Job demands-resources theory extended: Stress, loneliness, and care responsibilities impacting UK doctoral students’ and academics’ mental health. Studies in Higher Education.

[B93-behavsci-16-01439] Umerenkova A. G., Flores J. G. (2022). Experiencias y emociones sobre la procrastinación en alumnado universitario con diferentes niveles de riesgo académico. REOP-Revista Española de Orientación y Psicopedagogía.

[B94-behavsci-16-01439] Uyaroğlu A. K., Ergin E., Tosun A. S., Erdem Ö. (2022). A cross-sectional study of social media addiction and social and emotional loneliness in university students in Turkey. Perspectives in Psychiatric Care.

[B95-behavsci-16-01439] Vasquez A. R., Barco G. C., Porras F. R. E., Niño G. L. E. M. (2023). Validez y confiabilidad de la escala de rendimiento académico desde la percepción del alumno. Revista de Ciencias Sociales.

[B96-behavsci-16-01439] Vicary E., Kapadia D., Bee P., Bennion M., Brooks H. (2025). The impact of social support on university students living with mental illness: A systematic review and narrative synthesis. Journal of Mental Health.

[B97-behavsci-16-01439] Vieyra G. L., Barrera J. E. B., Peña J. V., Pérez B. M. R., Loeza B. M. L. (2024). Rendimiento académico en estudiantes de una universidad pública estatal del centro occidente de México. Estudios y Perspectivas Revista Científica y Académica.

[B98-behavsci-16-01439] Walsh B. E., Rottenberg J., Schlauch R. C. (2025). Why loneliness requires a multidimensional approach: A critical narrative review. Nature Mental Health.

[B99-behavsci-16-01439] Wang Y. (2015). Research on optimization of time concept and management strategy of post-90s university students. 2015 international conference on education technology, management and humanities science (ETMHS 2015).

[B100-behavsci-16-01439] Wang Y. W., Abebe I., Cruz T. E., Arevalo Avalos M. R., Sifat M., Abdelwahab S., Li T. (2024). College students’ coping, resilience, well-being, academic experiences, and help-seeking attitudes during COVID-19. The Counseling Psychologist.

[B101-behavsci-16-01439] Wenig V., Heinrichs K., Heumann E., Lehnchen J., Burian J., Deptolla Z., Stock C. (2025). Social-ecological factors associated with loneliness in university students: Results from the German cross-sectional StudiBiFra study. Frontiers in Psychiatry.

[B102-behavsci-16-01439] Wijbenga L., van der Velde J., Korevaar E. L., Reijneveld S. A., Hofstra J., de Winter A. F. (2024). Emotional problems and academic performance: The role of executive functioning skills in undergraduate students. Journal of Further and Higher Education.

[B103-behavsci-16-01439] Wolters C. A., Brady A. C. (2021). College students’ time management: A self-regulated learning perspective. Educational Psychology Review.

[B104-behavsci-16-01439] World Health Organization (2022). World mental health report: Transforming mental health for all (Informe).

[B105-behavsci-16-01439] Yani D. I., Chua J. Y. X., Wong J. C. M., Pikkarainen M., Shorey S. (2025). The effects of universal educational interventions in improving mental health literacy, depression, and anxiety among adolescents: A systematic review and meta-analysis. International Journal of Mental Health Nursing.

[B106-behavsci-16-01439] Zhang J., Meng J., Wen X. (2025). The relationship between stress and academic burnout in college students: Evidence from longitudinal data on indirect effects. Frontiers in Psychology.

